# Validation of a Rapid and Easy-to-Apply Method to Simultaneously Quantify Co-Loaded Dexamethasone and Melatonin PLGA Microspheres by HPLC-UV: Encapsulation Efficiency and In Vitro Release

**DOI:** 10.3390/pharmaceutics14020288

**Published:** 2022-01-26

**Authors:** Marco Brugnera, Marta Vicario-de-la-Torre, Vanessa Andrés-Guerrero, Irene Bravo-Osuna, Irene Teresa Molina-Martínez, Rocío Herrero-Vanrell

**Affiliations:** 1 Innovation, Therapy and Pharmaceutical Development in Ophthalmology (InnOftal) Research Group, UCM 920415, Department of Pharmaceutics and Food Technology, Faculty of Pharmacy, Complutense University of Madrid, Plaza Ramón y Cajal, s/n, 28040 Madrid, Spain; marcobru@ucm.es (M.B.); mvicario@ucm.es (M.V.-d.-l.-T.); vandres@ucm.es (V.A.-G.); ibravo@ucm.es (I.B.-O.); iremm@ucm.es (I.T.M.-M.); 2 Sanitary Research Institute of the San Carlos Clinical Hospital (IdISSC), San Carlos Clinical Hospital, Calle Profesor Martín Lagos, s/n, 28040 Madrid, Spain

**Keywords:** glaucoma, melatonin, dexamethasone, validation, HPLC-UV, PLGA, microspheres, co-delivery, encapsulation efficiency, in vitro release

## Abstract

This paper discusses the development and validation of a rapid method for the reversed phase HPLC-UV quantification of biodegradable poly(D,L-lactic-co-glycolic) acid (PLGA) microspheres co-loaded with two neuroprotective agents (dexamethasone and melatonin) (DX-MEL-MSs) to be intravitreally administered as a promising glaucoma treatment. The study was performed to validate two procedures that quantify the content of the two active substances entrapped into the polymer matrix during an encapsulation efficiency assay and the amount of drugs liberated over time during the in vitro release assay. The reversed-phase method allowed for the simultaneous determination of dexamethasone and melatonin, which were respectively detected at 240.5 and 222.7 nm. Chromatographic separation was performed using an Ascentis^®^ C18 HPLC Column (25 cm × 4.6 mm, 5 µm) with an isocratic mobile phase composed of methanol-water (70:30, *v*/*v*) with 1.0 mL min^−1^ flow rate. The two procedures were validated analytically in terms of system suitability testing, specificity, linearity, precision, accuracy, sensitivity, and robustness. Both the validated procedures were applied to characterize DX-MEL-MSs and were found appropriate to quantify the drug quantities encapsulated and estimate their release profile over 10 days. The validation study designed in this work can be helpful for planning any other protocols that refer to the quantification of PLGA based drug delivery systems.

## 1. Introduction

Glaucoma, diabetic retinopathy, age-related macular degeneration, and retinitis pigmentosa are some of the optic neuropathies that have a major impact worldwide and can lead to progressive and permanent blindness [1]. Among them, glaucoma is a chronic and multifactorial disease that is characterized by a progressive death of retinal ganglion cells (RGC), the group of axons that form the nerve fibre layer, hence inducing the loss of vision [2]. One of the main risk factors that trigger this pathology is the increase in intraocular pressure (IOP). Nowadays, the only evidence-based treatment for glaucoma consists precisely of lowering IOP by reducing the aqueous humour production, or enhancing its outflow through the trabecular meshwork, to consequently avoid adverse effects at the level of the posterior segment of the eye [3]. However, it has been demonstrated that the decrease of IOP does not ensure protection against progression of the disease and also that there are patients with normal IOP values suffering from glaucoma [4,5]. Whilst the pathogenesis of glaucoma remains incompletely understood, all patients diagnosed with this disease are distinguished by neurodegeneration, which is defined as the progressive malfunction and loss of retinal neurons. Neurodegenerative processes lead to RGC death and involve several molecular mechanisms, such as neuroinflammation, excitotoxicity, axonal transport damage, and oxidative stress [6,7]. As all these mentioned events have demonstrated effects that can interact and be triggered by different pathways, co-delivery and administration of different agents can represent an effective clinical approach to treat glaucoma [8,9]. For this reason, neuroprotective strategies, such as the use of antioxidant or anti-inflammatory agents in combination, can represent a promising therapy for this chronic disease.

Among other drugs, melatonin and dexamethasone have proved to confer positive influence against the onset and the progression of glaucoma. Melatonin (MEL) is an indolamine neurohormone physiologically secreted from the pineal gland and retinal photoreceptors. The antioxidant activity of MEL and its metabolites has been fully studied in the last years [10,11,12]. Due to its high quantity of polyunsaturated fatty acids and its direct contact to light, the retina is particularly prone to oxidative stress. MEL mainly acts as a radical scavenger; it produces glutamate neurotoxicity impairment and inhibits the nitrergic pathway so having a protective effect on the photoreceptors outer membranes [13,14]. MEL also has proved to have a direct effect on reducing IOP [15,16,17]. Dexamethasone (DX) is a corticosteroid, which has long been used as an anti-inflammatory agent to treat posterior segment diseases, and has been demonstrated to have neuroprotective properties [18,19].

Both DX and MEL need to achieve considerable concentrations in the retina to be therapeutically effective. Intraocular drug delivery devices can provide a continuous supply of active substances close to the retina during the long term and represent an ideal system designed for neuroprotectants in chronic neurodegenerative diseases [20,21]. In this study, biodegradable poly(D,L-lactic-co-glycolic) acid (PLGA) microspheres were elaborated as a novel drug delivery system for the co-delivery of DX and MEL to be intravitreally administered. Biodegradable PLGA microspheres can represent an alternative to repeated intraocular injections to avoid any consequent side-effects. Moreover, they disappear from the site of administration after delivering the drugs and can be suitable for personalized therapy, as they can be easily tuned for each patient [22]. In the literature, DX and MEL have been already used in combination. In particular, Pan et al. developed DX and MEL co-loaded PLGA nanoparticles to be topically administered at eye level [23]. This drug delivery device was characterized in terms of the drugs’ content and release with two different chromatographic methods by liquid chromatography tandem mass spectrometry (LC/MS). These methods were developed and described in a recent published study from our group by Arranz-Romera et al. [19]. In this publication, we reported that DX, MEL and coenzyme Q10 co-delivered through PLGA microspheres managed to reduce the loss of RGC and resulted in more effective treatment than the physical mixture of single-loaded formulations.

Despite the advantages of LC/MS methods, such as high sensitivity and precision, this equipment requires complex installations, extended run times and consequently the waste of large amounts of hazardous solvents that entails high monetary and ecological costs. The purpose of this work was to validate a more straightforward and routine method to characterize PLGA microspheres co-loaded with DX and MEL (DX-MEL-MSs), as a new intraocular drug delivery system. Two procedures that share the same chromatographic conditions were developed and fully validated to simultaneously quantify DX and MEL with two different aims. The first procedure was developed for the simultaneous quantification of active substances loaded into microspheres for the encapsulation efficiency assay; the second one was created to quantify drugs released from microspheres during the in vitro release assay [24,25,26]. The in vitro release assay shows kinetics of the active substances liberated over time in a buffered release medium emulating physiological conditions [27]. The offered analytical method has been established using a high performance liquid chromatography (HPLC) technique coupled with ultraviolet (UV) detection and is revealed as fast and cost-effective in routine pharmaceutical analyses. Although evidence has been presented for the feasibility of DX and MEL quantification, several methods lacking simultaneous determination of the two mentioned compounds, or with longer retention times, have been described [28,29,30]. To the authors’ knowledge, there is no reversed phase HPLC-UV validated method in the literature for the quantification of DX and MEL simultaneously with a short analysis time that involves a reduction in solvent consumption.

## 2. Materials and Methods

### 2.1. Chemicals

Melatonin (M5250-5G) and dexamethasone (D1756-5G) were purchased from Sig-ma-Aldrich (Madrid, Spain). Poly(D,L-lactic-co-glycolic) (PLGA) acid 50:50 (D170500523; Resomer^®^ 503; 24,000–38,000 g/mol) was purchased from Evonik Industries (Essen, Ger-many). Sodium chloride was obtained from Merck (1.06404.1000, Merck KGaA, Darmstadt, Germany). Potassium di-hydrogen phosphate (131509.1211), di-sodium hydrogen phosphate 12-hydrate (131678.1211), polyvinyl alcohol 72,000 g/mol (A2255,0250), methanol (221091.1612), ethanol (221086.1612), acetonitrile (221881.1612) and dichloromethane stabilized with 20 ppm of amylene (361254.1612) were acquired from PanReac AppliChem (Barcelona, Spain). All solvents cited were HPLC grade. Water was purified from a Milli-Q^®^ filtration system (Millipore Corporation, Billerica, MA, USA). Acetone and all other chemicals were reagent grade and were used as received.

### 2.2. Equipment

The HPLC system was composed of a Separation Module Waters^®^ Alliance 2695 coupled with a Waters^®^ Photodiode Array 2996 detector, an on-line degasser and a Waters^®^ 186001863 HPLC Column Heater (Barcelona, Spain). Empower 3^®^ was the chromatography software used for collecting and processing data. All the analyses were carried out using an Ascentis^®^ C18 HPLC Column (25 cm × 4.6 mm, 5µm) purchased from Sigma-Aldrich (Madrid, Spain) as stationary phase. Vortex D-051 was acquired from Dinko (Barcelona, Spain) and used for the preparation of PLGA microspheres and encapsulation efficiency assay. A Micro 220R (Hettich, Aizarnazabal, Guipuzcoa, Spain) was the centrifuge used in the in vitro release assay, while a Universal 32 (Hettich, Aizarnazabal, Guipuzcoa, Spain) was the centrifuge employed in the encapsulation efficiency assay. The Memmert^®^ Shaking Bath (WNB 29) was acquired from Memmert GmbH (Schwabach, Germany) and used in the in vitro release assay. Both a Sonorex Digiplus DL 510 H (Bandelin, Berlin, Germany) and a Sonicator XL2020 (Heat Systems Inc., Farmingdale, NY, USA) were used in the elaboration of PLGA microspheres. A Polytron^®^RECO (Kinematica, GmbHT PT3000, Lucerna, Switzerland) was the homogenizer used in the preparation of PLGA microspheres.

### 2.3. Chromatographic Conditions

The column was maintained at 45.0 ± 0.2 °C throughout the analysis. Flow rate was set at 1.0 mL min^−1^ and the injection volume was 10 µL. The composition of the mobile phase was methanol (MeOH)- Milli-Q^®^ water (70:30, *v*/*v*). The isocratic elution was monitored for 8.00 min. DX was detected by the UV diode array detector at 240.5 nm whereas MEL monitoring wavelength was set at 222.7 nm. Both compounds were detected at their maximum absorbance wavelength [31,32]. Samples were kept at 4.0 ± 0.5 °C in the carousels during the analysis. Both the analytical methods developed used the same chromatographic conditions.

### 2.4. Elaboration of PLGA Microspheres

PLGA microspheres (MSs) containing the active ingredients, DX and MEL, in a ratio 1/1:10 *w*/*w*, were manufactured using an oil/water emulsion solvent extraction-evaporation technique modifying the procedure described by Arranz-Romera et al. [19]. First, PLGA (400 mg) was dissolved in 0.7 mL of dichloromethane (DCM) by vortex mixing. Afterwards, a mixture of 40 mg DX and 40 mg MEL was ground in a mortar and added to the previous solution together with ethanol (25% *v*/*v*) [33]. The organic mixture was sonicated in an ice-water bath for 5 min and then agitated with a sonication probe for 1 min at 4 °C. A solution of 5 mL of aqueous polyvinyl alcohol (PVA) (2% *w*/*v*) was emulsified with the phase described above by using the homogenizer set at 6000 revolutions per minute (rpm) for 1 min. The resulting emulsion was then transferred to the maturation phase, consisting of 100 mL of PVA solution in water (0.1% *w*/*v*), and constantly stirred at room temperature for 3 h to remove the organic solvents by evaporation. Afterwards, the formed MSs were washed with Milli-Q^®^ water to remove any PVA or solvent traces and then separated into one granulometric fraction (38–20 µm) using two sieves (mesh size: 38 and 20 µm). Once the elected particle size was obtained, MSs were lyophilized (freezing: −60 °C/15 min; drying: −60 °C/12 h/0.1 mBar) and stored at −30 °C under dry conditions until required. The same exact procedure was followed to prepare unloaded MSs (blank-MSs).

### 2.5. Test Sample Preparation

#### 2.5.1. Encapsulation Efficiency Assay

**Figure 1:**
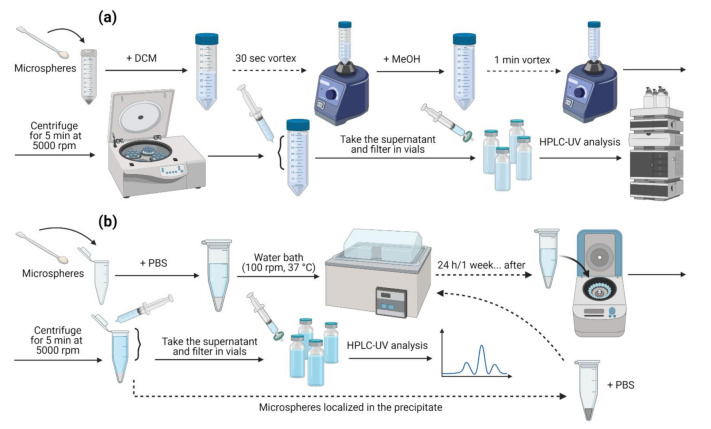
Schemes representing how drug extractions were performed for (**a**) EE and (**b**) IVR assays.

The drug content loaded into the pharmaceutical microsystems was determined by HPLC-UV following the method conditions previously described. Briefly, 5 mg of MSs were weighted and vortex mixed for 30 s in 2.5 mL of DCM to reach a complete dissolution. Then, 6 mL of MeOH were added to the solution to promote polymer precipitation and the extraction of the active substances. After vortex shaking for 1 min, the samples were centrifuged at 5000 rpm for 5 min at 20 °C. Next, the methanolic supernatant was withdrawn and diluted 1:5 (*v*/*v*) in MeOH. Then, samples were filtered using 0.22 µm pore polyamide (nylon) syringe filters (JNY022013N, Filter-Lab^®^, Barcelona, Spain) before being subjected to HPLC-UV analysis to measure the content of the active substances. The assay is schematized in Figure 1a below.

The encapsulation efficiency (EE) refers to the amount of drug successfully entrapped into the drug delivery device [34]. In this case, DX and MEL contents were calculated as the ratio between the actual drug amount and the total added during the elaboration of the microspheres. All samples were analysed in duplicate and the results were reported as the average ± standard deviation (SD).

#### 2.5.2. In Vitro Release Assay

The profiles of the release of the active substances from MSs were determined by suspending 5 mg of MSs in 2 mL of phosphate buffer solution (PBS) (pH = 7.4) maintained at 37 °C under constant agitation (100 rpm) in a water shaking bath (simulating physiological conditions). At certain time intervals, all the volume added were withdrawn and the volume of liquid was replaced with fresh release medium and placed again under shaking. The in vitro release (IVR) test samples were submitted to the centrifugation process at 5000 rpm for 5 min at 20 °C. The drug content in the filtered supernatants (filtration through 0.22 µm nylon syringe filters) was analysed by HPLC-UV as outlined above.

Released quantities of DX and MEL were estimated as the drug amount present in the release media between each sampling with respect to the total encapsulated. Release profiles were analysed in triplicate and the results were expressed as the average ± SD. This assay is also represented in Figure 1b below.

### 2.6. Standard Solutions Preparation

The elaboration of the stock standard solutions followed a specific protocol for each analysis, encapsulation efficiency and in vitro release, while the conditions for the analytical methods were kept the same to optimize laboratory work.

#### 2.6.1. Encapsulation Efficiency Assay

Stock standard solutions for the determination of drug entrapment (SS-EE) were freshly prepared by dissolving 1 mg of both DX and MEL in 20 mL of MeOH to reach a concentration of 50 µg mL^−1^ for the active substances. Working solutions (WS-EE) containing DX (2.5–50 µg mL^−1^) and MEL (2.5–50 µg mL^−1^) were prepared by diluting SS-EE with MeOH.

#### 2.6.2. In Vitro Release Assay

Stock standard solutions to study the release profile in vitro (SS-IVR) were made by dissolving 20 mg of DX and MEL in 10 mL of MeOH. Hence, this solution was 1:100 (*v*/*v*) diluted in PBS to reach a final concentration of 20 µg mL^−1^ for both DX and MEL. Starting from this SS-IVR, DX (1–20 µg mL^−1^) and MEL (1–20 µg mL^−1^) working solutions (WS-IVR) were obtained by successive dilutions with PBS.

### 2.7. Validation Study

The validation study described below includes two test sample procedures, procedure A for the encapsulation efficiency quantification and procedure B for the in vitro release assay determination. Both approaches were validated according to the International Conference on Harmonization (ICH) Topic Q2 (R1) (“Validation of analytical procedures: text and methodology”; CPMP/ICH/381/95) and considering Food and Drug Administration (FDA) guideline (“Validation of chromatographic methods”) [35,36]. The procedures were validated in terms of system suitability testing, selectivity, linearity, precision (repeatability and intermediate precision), accuracy, sensitivity, and robustness.

#### 2.7.1. System Suitability Testing

System suitability testing was evaluated for both analytical procedures by analysing the resolution between peaks, injection repeatability, tailing factor and theoretical plate number according to FDA guideline [37]. Six replicate injections of the working solution (WS-EE) at 50 µg mL^−1^ for DX and MEL, and six replicates of the working solution (WS-IVR) at 10 µg mL^−1^ for DX and MEL, were assessed.

#### 2.7.2. Specificity

This test was executed to clearly distinguish DX and MEL chromatographic peaks in the presence of other components of the MSs, like PLGA or PVA remnants, by checking interference peaks and comparing with DX and MEL estimated retention time and the corresponding UV spectra. For the procedure A (to determine EE), the specificity was performed analysing three different samples of PLGA as a control, blank-MSs alone, blank-MSs in combination with MEL at 50 µg mL^−1^ (the highest concentration used for linearity test), blank-MSs with DX at 50 µg mL^−1^ and blank-MSs with both DX and MEL concentrations of 50 µg mL^−1^, for the two substances. Specificity for procedure B (to determine the IVR profile) was assessed by analysing triplicates of PLGA (raw material) as a control, blank-MSs alone, blank-MSs with a working solution of MEL in PBS at 10 µg mL^−1^, blank-MSs with a working solution of DX at 10 µg mL^−1^ and blank-MSs with both DX and MEL at 10 µg mL^−1^ for the two analytes.

#### 2.7.3. Linearity

Linearity was determined by analysing solutions at seven different concentrations in the range from 2.5 to 50 µg mL^−1^ for procedure A, and from 1 to 20 µg mL^−1^ for procedure B, derived respectively from three different SS-EE and SS-IVR solutions freshly prepared each day for three consecutive days. For the two procedures, the mentioned ranges were selected according to the theoretical amounts of drugs added during the elaboration of DX-MEL-MSs and their expected in vitro release, respectively. Three regression lines were analysed for each assay and each working solution was injected in triplicate. The regression lines were constructed by plotting peak areas against concentrations and calculated separately for procedures A and B. Points with linearity should fit on a straight line. They should be evaluated using the ordinary least squares method and slope, intercept and correlation coefficient (R > 0.999) were determined to ensure linearity.

#### 2.7.4. Precision

Precision of the methods was determined in terms of repeatability (intra-day precision) and intermediate precision (inter-day) by analysing three levels of concentration: 5, 20 and 40 µg mL^−1^ for the procedure A (encapsulation assay quantitation), and 2.5, 7.5 and 15 µg mL^−1^ for the procedure B (in vitro release analysis). Each level was assessed in sextuplicate (*n* = 6) within a single day (repeatability) and on three different days, for intermediate precision. Results were evaluated by recovery percentages and precision was expressed in terms of relative standard deviation (RSD).

#### 2.7.5. Accuracy

The validation of accuracy was carried out at three concentration levels for both analytical procedures developed. The accuracy for the procedure A was determined at 5, 20 and 40 µg mL^−1^, while for the procedure B, the concentrations evaluated were 2.5, 7.5 and 15 µg mL^−1^. The experiments were conducted in sextuplicate for each level on the same day and on three consecutive days. Samples were freshly prepared for each analysis day. Results for accuracy were reported as recovery percentages and were determined as the difference between the average and the theoretical value (expressed in %) together with the confidence intervals.

#### 2.7.6. Sensitivity

Sensitivity was tested in terms of limit of detection (LOD) and limit of quantification (LOQ). The LOD was estimated as the lowest concentration that the analytical procedures could reliably detect and it was calculated by gradually diluting stock solutions to reach the lowest concentration assayed for each drug, DX and MEL. The LOQ was the lowest concentration of active substance that could be quantified with acceptable precision and accuracy. According to IUPAC criteria, LOD and LOQ were calculated using the standard error of the intercept (σ) obtained by the linearity test divided by the slope (m) of the corresponding regression line using Equation (1) for LOD and Equation (2) for LOQ [38].

LOD = (σ/m)·3.3(1)

LOQ = (σ/m)·10(2)

Sensitivity was experimentally evaluated for the analytical procedure B, to quantify drugs during the in vitro release assay, as it is the approach that would be employed for the analysis of lowest concentrations. Sensitivity was evaluated by analysing six working solutions at a calculated LOQ concentration freshly prepared each day for three consecutive days. Results were expressed as mean (µg mL^−1^) ± RSD (%).

#### 2.7.7. Robustness

Robustness is a test that shows the consistency of the analysis with respect to deliberate modifications to method parameters. To determine robustness, slight parameter variations in the chromatographic conditions were made, such as composition of the mobile phase (68 and 72% of MeOH), column oven temperature (43 and 45 °C) and detection wavelength for the two active substances (±2 nm for both DX and MEL). Moreover, a severe change in injection volume (5 and 15 µL) was performed to check method robustness in aggressive situations. Six replicate injections of solutions of DX-MEL at 50 µg mL^−1^ in MeOH and solutions at 10 µg mL^−1^ in PBS, procedures A and B, respectively, were analysed by changing the parameters described above one by one. The effect of all these variations on drug retention time, peak symmetry and area responses was evaluated. Drug extraction procedure from MSs, like centrifugation time that was increased from 5 to 30 min by evaluating DX and MEL recoveries every 5 min, was also made to determine robustness. Four replicates of EE and IVR test samples were analysed to compare different centrifugation times for drug extraction from MSs and optimize the sample treatment. Additionally, method robustness was also determined by analysing WS-EE and WS-IVR after introducing major changes, such as a different analyst and different chromatographic equipment. Six replicates of three concentration levels, 5, 20 and 40 µg mL^−1^ for the procedure A, and 2.5, 7.5 and 15 µg mL^−1^ for procedure B, were analysed by two analysts on different days. The same solutions were analysed through HPLC line in a Waters^®^ Acquity Arc Bio UHPLC paired with a Waters^®^ Photodiode Array 2998 detector with the same stationary phase and chromatographic conditions in two occasions. Data related to the application of different analysts and instruments were expressed as average recovery percentages and RSD (%), the retention time of peaks and their symmetry were also reported.

### 2.8. Statistical Analysis

The experimental results were expressed as averages ± SD and RSD (%) were indicated when possible. A probability value lower than 0.05 (*p*-value < 0.05) was considered statistically significant. Statgraphics Centurion 19© (Statgraphics Technologies, Inc., The Plains, VA, USA) software was used for statistical determinations.

## 3. Results and Discussion

An easy-to-apply method, composed of two fast procedures for test sample preparation, was developed, with the aim to concurrently quantify DX and MEL for encapsulation efficiency and in vitro release assays in a single run, in a simple way, without excessive costs and in the shortest possible time, to allow the analysis of as many samples as possible with a low solvent consumption. The active ingredients were encapsulated in a new formulation of biodegradable microparticle systems to be intravitreally injected for the treatment of glaucoma. The analytical method was applied to the simultaneous quantification of DX and MEL encapsulated in the microparticulate formulations, which were extracted using MeOH (procedure A) and the in vitro co-delivery of these drugs from the microspheres in PBS as a release medium (procedure B).

### 3.1. Validation Procedure

Prior to the validation procedures, several experimental tests were performed to optimize the chromatographic conditions. MeOH and Milli-Q^®^ Water were selected as compatible solvents with the ones used for encapsulation efficiency and the in vitro release assays under isocratic conditions, thus isocratic elution allowed well-separated and defined peaks to be obtained. Composition of the mobile phase and flow rate were changed to obtain well resolved DX and MEL peaks in a short time. The initial tested ratios of MeOH and Milli-Q^®^ Water, 70:30 (*v*/*v*) and 50:50 (*v*/*v*), were assayed at different flow rates, from 1.00 to 1.25 mL min^−1^ and oven temperatures, from 30 °C to 50 °C. Finally, the mobile phase was constituted by a 70:30 (*v*/*v*) MeOH:Water mixture, at 1 mL min^−1^ and 45 °C oven temperature. The detection wavelengths for the simultaneous analysis of DX and MEL were 240.5 nm and 222.7 nm, respectively. Under these chromatographic conditions, the obtained DX and MEL peaks were defined, well resolved and free from tailing and had a nominal difference in retention time of 1.7 min between them.

#### 3.1.1. System Suitability Testing

System suitability testing is required to ensure the reliability and consistency of the HPLC methods developed. The results obtained after injecting six replicates showed that the parameters evaluated complied with requirements established. The DX and MEL peaks had a proper shape and were repeatedly retained at 4.7 and 2.9 min, respectively, with RSD (%) of the recorded retention time (tR) < 0.25. Moreover, the width (tW) of the DX and MEL peaks was indicated. Both peaks were well-separated showing a resolution between peaks (Rs) > 3 (complying with the specification established Rs > 2) and the injection repeatability (IR) did not exceed 1.5 value (limit value < 2) that indicated an excellent repeatability of replicate injections. The tailing factor (T) for both peaks was always under 1.5 denoting a good peak symmetry (acceptance value < 2), whereas N (theoretical plate number) was considerably higher than 2000 (acceptance limit > 2000), showing that the column was efficient in performing the separation between the DX and MEL peaks for both methods developed. The results are reported in Table 1.

#### 3.1.2. Specificity

**Figure 2:**
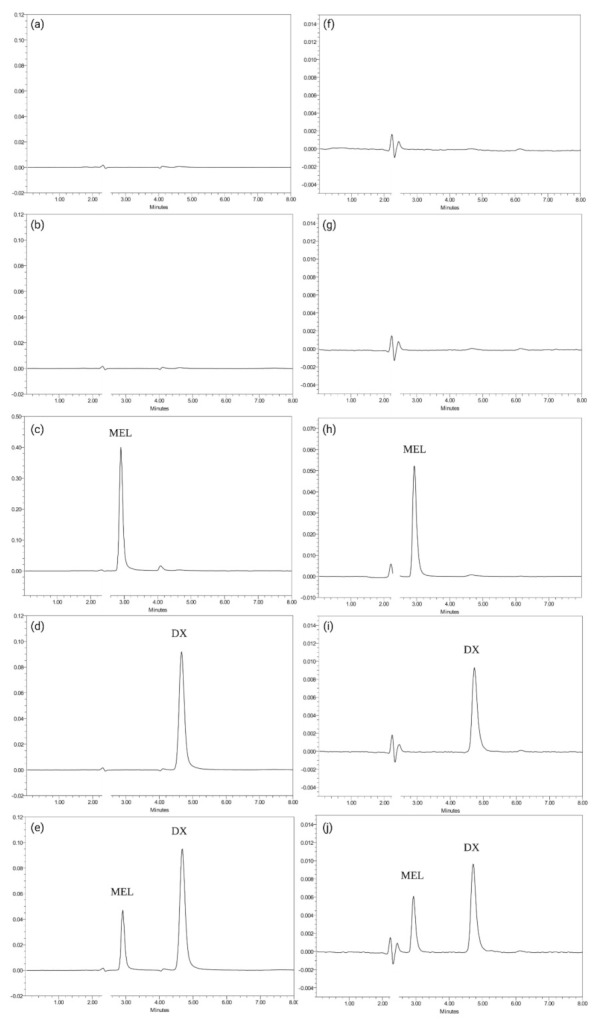
Chromatograms obtained for procedure A (for encapsulation efficiency quantification) (**a**–**e**) and for procedure B specificity (for in vitro release analysis) (**f**–**j**) following EE and IVR test sample preparations. The x-axis represents retention time whereas the y-axis represents absorbance units. (**a**) PLGA; (**b**) blank-MSs alone; (**c**) blank-MSs with MEL in MeOH at 50 µg mL^−1^; (**d**) blank-MSs with DX at 50 µg mL^−1^; (**e**) blank-MSs with both DX and MEL at 50 µg mL^−1^; (**f**) PLGA; (**g**) blank-MSs alone; (**h**) blank-MSs with MEL in PBS at 10 µg mL^−1^; (**i**) blank-MSs with DX at 10 µg mL^−1^; (**j**) blank-MSs with both DX and MEL at 10 µg mL^−1^.

Specificity was assessed to distinguish between the active substances and the compounds present in the matrix of the microparticulate systems. Specificity was evaluated for procedures A and B by following EE and IVR test sample preparations, respectively. Chromatograms of solutions containing DX and MEL and all potential components, for instance PLGA, DCM or PBS, were compared with responses to blank solutions. Chromatograms performed for specificity are represented in Figure 2 below. All chromatograms shared a mobile phase front at around 2.2 min. MEL represented the first peak in the chromatograms (2.9 min), whereas DX was eluted in a second moment (4.7 min). Blank MSs chromatograms spiked with a solution of MEL and DX at 50 µg mL^−1^ (Figure 2e for procedure A) and at 10 µg mL^−1^ (Figure 2j for procedure B) showed no relevant peaks, but the MEL and DX peaked at their corresponding retention time. Regarding both procedures, neither PLGA, nor blank-MSs, showed interferences with the retention times of the two active substances. All chromatograms related to procedure A specificity showed a small peak around 4.0 min, which was linked to DCM because, following EE assay procedure, PLGA was solubilized in DCM and then precipitated by MeOH, ensuring that the polymer was not present during chromatographic analysis. On the other hand, it is assumed that PLGA underwent hydrolysis reaction during IVR assay leading to the formation of lactic acid and glycolic acid, which resulted in no interference on the chromatograms [39]. In the same way, any salts constituting PBS did not interfere in the chromatograms of IVR assay.

#### 3.1.3. Linearity

The linearity results acquired and their statistical analysis are reported in Table 2. Regarding procedure B (for in vitro release quantitation), linearity was evaluated across a lower and narrower range of concentrations (1–20 µg mL^−1^) compared to procedure A (2.5–50 µg mL^−1^), as lower and narrower concentrations of DX and MEL should be quantified in the release medium throughout the release study.

First, the homoscedasticity of the data acquired was verified for both analytical procedures by Bartlett’s test. Since there was no statistical significance (*p*-value > 0.05) for MEL and DX along the concentration ranges (2.5–50 µg mL^−1^ and 1–20 µg mL^−1^) for the variances of the residuals (homoscedasticity), linearity results were explained by a normal distribution and least squares method was used to fit the regression line.

Then, the calibration curves constructed were linear in the range of concentrations for procedure A (2.5–50 µg mL^−1^) and procedure B (1–20 µg mL^−1^) (*p*-values < 0.05) with a correlation coefficient (R) > 0.999, for both the active substances confirmed by statistical analysis (ANOVA). Linearity was also demonstrated by evaluating statistical significance in the slope (*p*-value < 0.05) and intercept (*p*-value > 0.05) (Student’s *t*-test).

#### 3.1.4. Precision

Precision of the methods was determined in terms of the repeatability and the intermediate precision, by calculating recovery percentages from six replicates at three concentration levels: 5, 20 and 40 µg mL^−1^ for procedure A and 2.5, 7.5 and 15 µg mL^−1^ for procedure B, pleasing ICH criteria. Recovery percentages are reported in Table 3 below [37].

The precision results obtained and their statistical analysis are reported in Table 4. As recoveries (%) were not statistically different (*p*-value > 0.05 for all situations by Levene’s test), data were evaluated as a whole and a unique RSD (%) for the three levels were presented both for intra-day and inter-day precision. As for both procedures, the intermediate precision (inter-day) values were lower than the repeatability (intra-day) ones, the latter is considered as the precision of the analytical method. As it is shown in Table 4, the ANOVA *F*-test’s probability values (*p*-value > 0.05) indicated that there was no significant difference between the three different days of analysis. Intra-day and intermediate RSD (%) of MEL and DX never exceeded 1.5% for the two analytical methods (acceptance limit RSD (%) < 2) thus, indicating the high precision of the procedures developed.

#### 3.1.5. Accuracy

Accuracy was calculated by comparing the measured values (recovery percentages) for MEL and DX to the theoretical concentrations (100%). The accuracy results and the statistical analysis are reported in Table 5. Accuracy was evaluated by examining six replicates at three concentration levels freshly prepared on three consecutive days. The recovery results showed that data were homogeneous (*p*-value > 0.05) (Levene’s test) and the concentration levels were not statistically different (*p*-value > 0.05) (ANOVA test) for both analytical methods. The recovery (%) of MEL was between 99.8–100.4% and 99.7–100.3%, while DX was between 100.2–100.9% and 99.6–100.4% for procedures A and B, respectively. All data obtained were within the FDA specification (range between 97.0–103.0%) and therefore, it was possible to simultaneously determine MEL and DX with accuracy and quantify the drugs in the EE and IVR assays by using procedure A and B, respectively [40].

#### 3.1.6. Sensitivity

The sensitivity of the method was estimated by calculating LOD and LOQ according to Equations (1) and (2), respectively. Regarding procedure A, LOD was equal to 0.25 µg mL^−1^ for MEL and 0.31 µg mL^−1^ for DX. As regards procedure B, the LOD obtained were 0.11 and 0.13 µg mL^−1^ for MEL and DX, respectively. The LOQ for the encapsulation efficiency assay was calculated as 0.76 µg mL^−1^ for MEL and 0.95 µg mL^−1^ for DX. Concerning the in vitro release assay, the LOQ was 0.33 µg mL^−1^ for MEL and 0.39 µg mL^−1^ for DX. The LOD and LOQ for the two active substances were below the concentration range used for the analytical methods developed.

The sensitivity of the procedure B has been checked experimentally for MEL and DX by analysing six working solutions, freshly prepared each day on three consecutive days, at calculated LOQ concentrations. There was no significant difference between replicates (*p*-value > 0.05 for both agents). The results (Table 6) showed that mean LOQs were 0.34 ± 0.03 µg mL^−1^ for MEL and 0.41 ± 0.02 µg mL^−1^ for DX with repeatability and intermediate precision RSD (%) that accomplished the acceptance limit (RSD < 10%), according to FDA guidelines [41]. Thus, this method can simultaneously measure MEL and DX at low concentrations, and it was advantageous for the quantification of the agents during the sustained in vitro release from the microparticles.

#### 3.1.7. Robustness

Robustness was determined by evaluating the impact of deliberated changes in the chromatographic conditions on peak retention time, peak symmetry, and recovery percentages. The detection wavelengths of 222.7 ± 2.0 nm and 240.5 ± 2.0 nm referred to MEL and DX, respectively. The robustness results are reported in Table 7. The slight changes examined did not significantly influence neither retention time, for which RSD is well within the proposed acceptance criteria (RSD < 5%), nor peak symmetry (pS) that was in the acceptance limit (RSD < 2%) [37]. Modifying the composition of the mobile phase resulted in variation in tR, which did not impact recovery percentages and should be precautionary controlled in routine analyses. All the three modifications produced average recovery percentages, which ranged in the interval 98.0–102.0% for both MEL and DX in the two procedures studied, for which RSD% was also included with acceptable 2% variation. Both procedures A and B can be considered robust under the mentioned changes in chromatographic conditions within the protocol.

Changing the injection volume did not significantly affect either tR (RSD < 0.3%), or pS. The area responses, as a function of injection volume, fitted to a linear regression (R > 0.999), maintained proportionality to changes in the injection volume with a ratio of the areas of 0.5 and 1.5, at 5 µL and 15 µL, respectively. Critical changes, instead of small modifications, in the chromatographic conditions were performed as a worst scenario and the results have ensured robustness for the analytical procedures proposed. Results are reported in Table 8.

**Figure 3:**
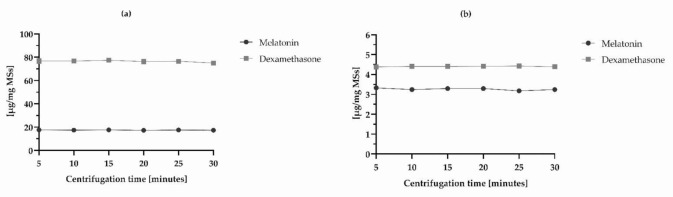
Optimization of centrifugation time during test sample preparation of (**a**) EE and (**b**) IVR assays.

The modification of centrifugation time of the samples for EE and IVR quantitation was performed in four replicates to optimize drug extraction procedure and ensure their robust quantification. The centrifugation time was increased from 5 to 30 min successively, and the DX and MEL recoveries (%) were calculated every 5 min. Regarding the in vitro release assay, data were referred to 24-h release. Results (Table 9) demonstrated that there was no statistical significant difference between the measurements obtained over the six different centrifugation times, ergo “5 min” was the parameter selected as it was meaningless considering higher times in absence of significant values. Precisely, RSD of all determinations obtained by changing centrifugation times complied with acceptance criteria (RSD < 5%) for the two active ingredients of both the assays [40]. These results suggested that the DX and MEL extraction recoveries were reliable and reproducible (Figure 3).

Additionally, three concentration levels of DEX and MEL from WS-EE and WS-IVR were analysed over two days using the established chromatographic conditions but changing the analyst and the separation system. Outcomes related to these changes are reported in Tables S1 and S2 in Supplementary Material. The results show that the RSD (%) obtained for both active substances, DEX and MEL, were < 2% in low, medium and high concentration levels for the procedures A and B. In the same way, tR and pS were within the acceptance limit.

### 3.2. Methods Applicability

**Figure 4:**
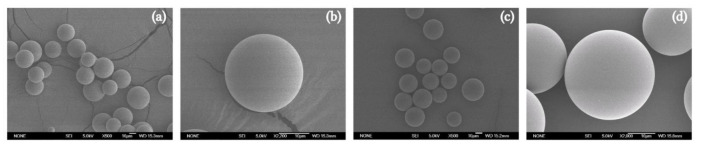
SEM images (SEM; Jeol, JSM-6335F, Tokyo, Japan) of (**a**,**b**) blank-MSs and (**c**,**d**) DX-MEL-MSs prepared using oil/water emulsion solvent extraction-evaporation technique. Each powder was spread on a double-sided carbon tape installed on an aluminium stub to be gold-coated under vacuum (K550X ion sputter, Emitech, Ashford, UK) for 2 min at 25 mA. SEM images were recorded at an acceleration voltage of 5.0 kV and (**a**,**c**) ×500 and (**b**,**d**) ×2000 magnification respectively for group and individual pictures.

The validated method was applied to characterize DX-MEL-MSs elaborated using an oil/water emulsion solvent extraction-evaporation technique. The manufactured MSs were quantified according to the described procedures for test sample preparations of EE and IVT assays, respectively. MSs were sieved in order to obtain a particle size in the range between 38–20 µm, that is suitable to be administered intravitreally as suspensions using conventional 30-gauge needles without the requirement of ocular surgery [42]. With the purpose of verifying their manufacture, the morphology of the MSs were evaluated by scanning electron microscopy (SEM) (Figure 4). Blank-MSs (Figure 4a,b) and DX-MEL-MSs (Figure 4c,d) exhibited smooth and spherical shape with absence of any crystals on the surface. Moreover, DX-MEL-MSs were analysed in terms of the encapsulation efficiency of the two active entrapped substances and their release in a buffered media applying procedure A and procedure B, respectively. Six replicates of DX-MEL-MSs were characterized following the test sample preparation technique.

**Figure 5:**
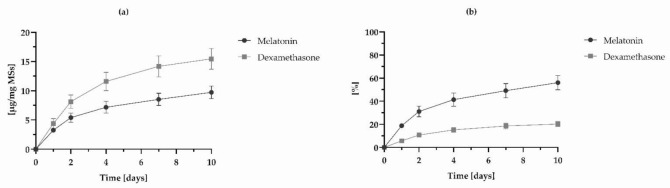
In vitro release profiles of DX and MEL from MSs expressed as (**a**) the quantity of drug in an established amount of MSs [µg drug mg MSs^−^^1^] and (**b**) in percentage. Data points are reported with their SD (*n* = 6).

The average encapsulation efficiencies found for DX and MEL were 91.25 ± 0.69% and 20.75 ± 0.25%, respectively, and drug loading, were 76.33 ± 0.75 µg mg MSs^−1^ for DX and 17.36 ± 0.20 µg mg MSs^−1^ for MEL. To prove the consistency of the analytical procedure B, DX-MEL-MSs controlled release was evaluated over 10 days. All the data related to the IVR assay referred to the extraction of samples at 24 h, also called burst release time, 2, 4, 7 and 10 days as the periods of time in which most of DX and MEL are delivered and which consist of a key feature of their release profile from MSs [43]. The co-loaded formulation release of DX was 5.74 ± 1.14% (4.38 ± 0.87 µg mg MSs^−1^) during the first 24 h and reached a total of 20.26 ± 2.34% (15.46 ± 1.79 µg mg MSs^−1^) after 10 days. Likewise, the MEL burst release was 18.82 ± 1.88% (3.27 ± 0.33 µg mg MSs^−1^) and the accumulated release quantity was equal to 56.01 ± 6.21% (9.72 ± 1.08 µg mg MSs^−1^) after 10 days. Figure 5 shows the release profiles of the co-loaded microparticles.

The MEL and DX peaks after extraction from MSs were well eluted and showed more similar characteristics than the peaks obtained from SS-EE and SS-IVR. The method developed, thus, was demonstrated to be rapid and effective for the simultaneous quantification of MEL and DX for EE and during in vitro release assays. Nevertheless, further studies will be required to understand the DX and MEL release profiles over a larger period of time. Moreover, future experiments are needed for the evaluation of DX and MEL MSs in contact with biological fluids and tissues that will provide valuable data about the feasibility of procedures A and B to analyse the active substances with a proper specificity.

## 4. Conclusions

The use of therapies that combine different active agents released from complex delivery systems have been explored for multifactorial and chronic diseases such as glaucoma. Therefore, the simultaneous quantitation of more than one drug by simple chromatography methods is of great interest for the development and characterization of new pharmaceutical platforms able to release drugs in a sustained manner.

A simple, rapid and easy-to-apply method was developed and fully validated to simultaneously quantify DX and MEL using reversed phase HPLC with UV detection. The two compounds were loaded in biodegradable PLGA microspheres for intravitreal administration as a neuroprotective treatment for glaucoma. One procedure (A) was proposed to quantify the content of the two active substances entrapped into the polymer matrix, the second one (B) was intended for the quantification of DX and MEL liberated over time during in vitro release assay. The two procedures differ for preparation of test samples but share the same chromatographic conditions. The method described was performed under isocratic conditions and was able to separate the two active substances with a proper resolution and in a relatively short run time that can be suitable for routine analyses. The two procedures were validated by means of system suitability testing, specificity, linearity, precision, accuracy, sensitivity, and robustness. All the mentioned tests satisfied ICH and FDA acceptance criteria. Both the validated approaches were applied to characterize DX-MEL-MSs and were found appropriate to simultaneously quantify both drugs encapsulated and estimate their release profile over 10 days. The validation study designed in this work focuses on the in vitro quantification of two active substances simultaneously and can be helpful for planning any other protocols that refer to the quantification of compounds loaded in PLGA based drug delivery systems.

## Figures and Tables

**Table 1:** System suitability testing average results respectively for the analytical procedures A and B. -RSD of the six measurements are reported in brackets.

	Procedure A (for EE Determination)		Procedure B (for IVR Quantitation)	
	50 µg mL^−1^		10 µg mL^−1^	
	MEL	DX	MEL	DX
tR	2.91 (0.24)	4.69 (0.24)	2.94 (0.13)	4.73 (0.11)
tW	0.65 (1.04)	0.63 (2.80)	0.59 (1.54)	0.73 (1.92)
Rs	3.42 (0.16)		4.39 (0.98)	
IR	0.11	0.21	0.42	0.45
T	1.21 (0.15)	1.20 (0.38)	1.46 (2.09)	1.34 (1.30)
N	3309.12 (1.96)	5029.93 (1.00)	4531.83 (2.49)	5732.29 (2.75)

**Table 2:** Linearity statistical parameters regarding both analytical methods for procedure A (EE quantitation) and procedure B (IVR assay) determination—*p*-values are reported in brackets.

	Procedure A		Procedure B	
	MEL	DX	MEL	DX
Slope	64,272.9	22,435.8	62,259.9	21,169.4
Standard error slope	185.52	80.76	202.99	82.05
Intercept	−6243.69	3504.68	−3275.71	916.62
Standard error intercept (σ)	4878.03	2123.61	2046.99	827.37
*t*-Statistic slope (*p*)	346.46 (0.000) *	277.80 (0.000) *	306.71 (0.000) *	258.01 (0.000) *
*t*-Statistic intercept (*p*)	−1.28 (0.2134)	1.65 (0.113)	−1.60 (0.124)	1.11 (0.280)
Correlation coefficient (R)	0.9999	0.9999	0.9999	0.9998
“Χ^2^” Bartlett’s test (*p*)	11.89 (0.064)	4.96 (0.549)	6.54 (0.365)	9.87 (0.130)
ANOVA *F*-test for regression (*p*)	120,032.96 (0.000) *	77,170.01 (0.000) *	94,070.56 (0.000) *	66,571.46 (0.000) *

* Statistical significance (*p*-value < 0.05).

**Table 3:** Results of intra-day and inter-day precision and accuracy expressed as recovery (%). All values inserted in the concentration (Conc) column are expressed in µg mL^−1^.

Procedure A							Procedure B						
Conc	MEL			DX			Conc	MEL			DX		
	Day 1	Day 2	Day 3	Day 1	Day 2	Day 3		Day 1	Day 2	Day 3	Day 1	Day 2	Day 3
5	100.78	99.79	101.70	101.73	100.67	102.44	2.5	100.90	99.11	100.11	101.63	101.24	98.46
	100.82	99.52	101.79	101.96	102.57	101.32		101.00	98.82	99.75	100.88	101.16	97.26
	100.32	100.86	99.09	98.49	100.86	102.37		99.95	101.52	98.89	99.02	99.90	99.17
	100.12	100.18	99.99	98.79	100.98	102.33		100.17	101.37	98.82	98.14	100.20	99.50
	99.83	100.58	98.07	102.08	99.35	99.11		98.80	99.04	100.74	100.13	97.47	101.25
	99.52	100.31	97.87	101.88	99.10	99.54		98.45	98.67	100.57	99.41	97.26	101.94
20	98.87	100.27	98.28	98.54	100.03	98.53	7.5	100.00	100.86	99.97	100.26	101.89	100.06
	98.57	100.32	98.57	98.68	99.95	98.71		100.10	101.00	99.83	100.17	101.75	100.18
	101.57	101.30	101.63	101.36	101.61	101.11		98.49	99.75	98.25	98.16	99.86	98.28
	101.70	100.75	101.10	101.70	101.80	101.09		98.37	99.79	98.41	98.38	99.78	98.38
	100.44	99.06	100.62	100.37	99.15	100.47		101.31	98.29	101.00	101.58	98.38	102.00
	100.15	98.90	100.01	100.59	99.22	100.16		100.95	98.22	101.11	101.95	99.25	101.69
40	100.58	98.12	100.64	101.33	99.87	101.92	15	100.80	99.47	101.05	99.87	100.04	101.05
	100.65	98.46	101.06	101.27	99.70	101.88		100.60	99.65	100.37	99.77	100.92	101.30
	100.69	100.91	100.85	100.99	100.60	100.50		98.29	101.26	100.90	101.62	101.27	99.22
	100.72	101.08	100.95	101.44	100.90	100.31		98.25	101.37	100.80	101.71	101.68	99.13
	98.49	101.19	98.68	99.59	101.77	98.36		101.27	100.92	99.43	98.82	99.99	100.02
	98.67	101.28	99.00	99.65	101.82	98.50		101.33	101.08	99.30	97.85	99.23	100.01

**Table 4:** Precision results and statistical values regarding both analytical methods employed for quantitation in the EE efficiency (procedure A) and IVR assay (procedure B)—*p*-values are reported in brackets.

	Procedure A		Procedure B	
	MEL	DX	MEL	DX
Average (recovery percentages)	100.10	100.54	99.97	99.99
RSD (%) repeatability	1.11	1.26	1.10	1.38
RSD (%) intermediate precision	1.09	1.24	1.08	1.36
“W” Levene’s test (*p*)	2.58 (0.085)	0.98 (0.381)	1.35 (0.269)	0.05 (0.950)
ANOVA *F*-test inter-day (*p*)	0.12 (0.889)	0.03 (0.971)	0.02 (0.983)	0.05 (0.955)

**Table 5:** Accuracy results and statistical values for the analytical methods developed—*p*-values are reported in brackets.

	Procedure A		Procedure B	
	MEL	DX	MEL	DX
Average (recovery percentages)	100.10	100.54	99.97	99.99
RSD (recovery percentages, %)	1.09	1.24	1.08	1.36
Confidence interval recovery percentages	99.80–100.40	100.20–100.88	99.68–100.27	99.62–100.36
“W” Levene’s test (*p*)	1.02 (0.369)	1.36 (0.265)	0.16 (0.852)	1.10 (0.341)
ANOVA *F*-test inter-day (*p*)	0.01 (0.987)	1.44 (0.248)	1.63 (0.206)	0.78 (0.465)

**Table 6:** The LOQ of DX and MEL for analytical procedure B to quantify agents during the in vitro release assay. All values inserted in the concentration and average columns are expressed in µg mL^−1^.

	MEL				DX			
Day	Conc	Average	SD	RSD (%)	Conc	Average	SD	RSD (%)
1	0.34	0.35	0.03	8.09	0.39	0.40	0.02	5.77
	0.32				0.37			
	0.33				0.42			
	0.34				0.43			
	0.40				0.39			
	0.35				0.42			
2	0.38	0.35	0.03	9.07	0.40	0.41	0.02	5.19
	0.33				0.42			
	0.37				0.39			
	0.39				0.43			
	0.32				0.44			
	0.32				0.39			
3	0.33	0.34	0.01	3.66	0.39	0.41	0.02	4.62
	0.35				0.42			
	0.33				0.41			
	0.35				0.43			
	0.32				0.38			
	0.33				0.40			
Average			0.34			0.41		
SD			0.03			0.02		
RSD (%)			7.41			5.22		

**Table 7:** Robustness results regarding changes in composition of the mobile phase, column oven temperature and detection wavelength for the active substances for both analytical methods.

		Parameter	Mobile Phase (% MeOH)			Column Oven Temperature (°C)			Detection Wavelength (nm)		
		Value	68	70	72	43	45	47	220.7 238.5	222.7 240.5	224.7 242.5
Procedure A	MEL	Average (recovery%)	99.44	100.54	101.47	98.99	100.84	101.63	98.40	100.10	99.18
		RSD (recovery%)	0.26	0.44	0.35	0.55	0.73	0.63	0.31	0.42	0.54
		tR (RSD%)	2.97 (0.21)	2.89 (0.21)	2.82 (0.19)	2.91 (0.18)	2.89 (0.17)	2.87 (0.23)	2.86 (0.21)	2.88 (0.16)	2.88 (0.16)
		pS (RSD%)	1.34 (1.11)	1.31 (1.44)	1.33 (1.27)	1.32 (0.48)	1.34 (1.54)	1.32 (0.70)	1.33 (1.09)	1.35 (0.96)	1.33 (0.68)
	DX	Average (recovery%)	99.36	100.53	101.06	98.66	100.90	101.74	99.74	100.10	99.14
		RSD (recovery%)	0.58	1.01	0.41	0.98	0.82	0.89	0.88	1.01	0.95
		tR (RSD%)	5.16 (0.15)	4.68 (0.13)	4.28 (0.10)	4.78 (0.09)	4.68 (0.14)	4.58 (0.15)	4.66 (0.09)	4.67 (0.13)	4.66 (0.13)
		pS (RSD%)	1.38 (0.73)	1.35 (0.69)	1.39 (1.43)	1.32 (0.37)	1.37 (0.98)	1.38 (0.42)	1.37 (0.87)	1.39 (0.75)	1.37 (0.57)
Procedure B	MEL	Average (recovery%)	98.13	100.42	99.13	98.76	99.78	101.14	98.90	100.34	98.53
		RSD (recovery%)	0.77	0.79	0.67	0.48	0.53	0.55	0.60	0.35	0.32
		tR (RSD%)	3.01 (0.24)	2.94 (0.22)	2.87 (0.24)	2.96 (0.22)	2.94 (0.15)	2.92 (0.21)	2.92 (0.24)	2.92 (0.23)	2.92 (0.23)
		pS (RSD%)	1.40 (1.90)	1.43 (1.75)	1.70 (1.86)	1.49 (1.81)	1.44 (1.78)	1.52 (1.40)	1.58 (1.71)	1.52 (1.91)	1.60 (1.77)
	DX	Average (recovery%)	99.56	100.74	98.85	98.87	99.56	101.56	98.32	99.94	98.86
		RSD (recovery%)	0.63	0.86	1.18	0.83	0.95	0.32	0.54	0.41	0.58
		tR (RSD%)	5.21 (0.16)	4.75 (0.15)	4.34 (0.17)	4.85 (0.14)	4.75 (0.06)	4.64 (0.14)	4.72 (0.18)	4.72 (0.18)	4.72 (0.18)
		pS (RSD%)	1.40 (1.77)	1.43 (1.73)	1.62 (1.05)	1.51 (1.98)	1.41 (1.82)	1.46 (1.76)	1.54 (1.63)	1.54 (1.60)	1.56 (1.57)

**Table 8:** Robustness results regarding injection volume changes for both analytical procedures. Area responses are expressed in AU·min.

			Injection Volume		
			5 µL	10 µL	15 µL
Procedure A	MEL	tR (RSD%)	2.90 (0.17)	2.91 (0.24)	2.90 (0.10)
		pS (RSD%)	1.26 (0.51)	1.21 (0.15)	1.15 (0.17)
		Area responses (mean ± RSD%)	1,615,543.83 ± 0.11	3,225,811.17 ± 0.11	4,836,931.33 ± 0.11
		*p*-value intercept (ANOVA)	0.072		
		*p*-value slope (ANOVA)	<0.001		
		Correlation coefficient (R)	>0.999		
	DX	tR (RSD%)	4.68 (0.11)	4.69 (0.24)	4.67 (0.08)
		pS (RSD%)	1.23 (0.19)	1.20 (0.38)	1.21 (0.36)
		Area responses (mean ± RSD%)	558,907.50 ± 0.13	1,121,940.50 ± 0.21	1,678,211.33 ± 0.11
		*p*-value intercept (ANOVA)	0.942		
		*p*-value slope (ANOVA)	0.002		
		Correlation coefficient (R)	>0.999		
					
Procedure B	MEL	tR (RSD%)	2.94 (0.31)	2.94 (0.13)	2.96 (0.14)
		pS (RSD%)	1.31 (1.80)	1.46 (1.89)	1.40 (1.72)
		Area responses (mean ± RSD%)	309,225.00 ± 0.17	623,427.333 ± 0.41	927,817.00 ± 0.42
		*p*-value intercept (ANOVA)	0.846		
		*p*-value slope (ANOVA)	0.006		
		Correlation coefficient (R)	>0.999		
	DX	tR (RSD%)	4.72 (0.20)	4.73 (0.11)	4.75 (0.15)
		pS (RSD%)	1.21 (1.21)	1.34 (1.30)	1.34 (1.83)
		Area responses (mean ± RSD%)	106,708.67 ± 0.44	211,243.50 ± 0.44	314,313.67 ± 0.29
		*p*-value intercept (ANOVA)	0.180		
		*p*-value slope (ANOVA)	0.003		
		Correlation coefficient (R)	>0.999		

**Table 9:** The robustness test regarding optimization of the parameter centrifugation time for test samples for the EE and IVR preparations. All values of concentration are expressed in µg mL^−1^ as average ± SD.

	Procedure A		Procedure B	
Conc	MEL	DX	MEL	DX
Average ± SD (*n* = 4) 5 min	17.59 ± 0.18	76.69 ± 2.10	3.33 ± 0.07	4.38 ± 0.10
Average ± SD (*n* = 4) 10 min	17.47 ± 0.30	76.69 ± 1.39	3.21 ± 0.11	4.41 ± 0.10
Average ± SD (*n* = 4) 15 min	17.62 ± 0.39	77.34 ± 1.89	3.29 ± 0.09	4.41 ± 0.11
Average ± SD (*n* = 4) 20 min	17.30 ± 0.44	76.30 ± 2.06	3.29 ± 0.08	4.41 ± 0.11
Average ± SD (*n* = 4) 25 min	17.52 ± 0.41	76.52 ± 1.51	3.18 ± 0.07	4.43 ± 0.09
Average ± SD (*n* = 4) 30 min	17.36 ± 0.50	75.08 ± 1.29	3.24 ± 0.06	4.38 ± 0.11
ANOVA *F*-test inter-group (*p*)	0.43 (0.822)	0.74 (0.603)	1.08 (0.405)	0.11 (0.988)
SD (all measurements)	0.36	1.69	0.09	0.09
RSD (all measurements) (%)	2.06	2.21	2.65	2.09

## Supplementary Materials

The following supporting information can be downloaded at https://www.mdpi.com/article/10.3390/pharmaceutics14020288/s1, Table S1: Robustness results expressed in average recovery percentages of six WS-EE and WS-IVR regarding changes in different analysts performing the test. Level line referred to low (5 and 2.5 µg mL^−1^ respectively for procedures A and B), medium (20 and 7.5 µg mL^−1^ respectively for procedures A and B) and high (40 and 15 µg mL^−1^ respectively for procedures A and B) concentration levels; Table S2: Robustness results expressed in average recovery percentages of six WS-EE and WS-IVR regarding changes in HPLC-UV equipment. Level line referred to low (5 and 2.5 µg mL^−1^ respectively for procedures A and B), medium (20 and 7.5 µg mL^−1^ respectively for procedures A and B) and high (40 and 15 µg mL^−1^ respectively for procedures A and B) concentration levels.

## Abbreviations

HPLC-UV	high performance liquid chromatography with ultraviolet detection
UV	ultraviolet
PLGA	poly(D,L-lactic-co-glycolic) acid
DX	dexamethasone
MEL	melatonin
MSs	microspheres
DX-MEL-MSs	dexamethasone and melatonin co-loaded PLGA microspheres
RGC	retinal ganglion cells
IOP	intraocular pressure
LC/MS	liquid chromatography tandem mass spectrometry
MeOH	methanol
DCM	dichloromethane
PVA	polyvinyl alcohol
blank-MSs	unloaded microspheres
EE	encapsulation efficiency assay
SD	standard deviation
PBS	phosphate buffer solution
IVR	in vitro release assay
SS	stock standard solution
SS-EE	stock standard solution for encapsulation efficiency assay
SS-IVR	stock standard solution for in vitro release assay
WS	working solution
WS-EE	working solution for encapsulation efficiency assay
WS-IVR	working solution for in vitro release assay
ICH	International Conference on Harmonization
FDA	Food and Drug Administration
RSD	relative standard deviation
LOD	limit of detection
LOQ	limit of quantification
tR	retention time
tW	peak width
Rs	resolution between peaks
IR	injection repeatability
T	tailing factor
N	theoretical plate number
pS	peak symmetry
SEM	scanning electron microscopy
