# Supplementary material for: Validation of a Rapid and Easy-to-Apply Method to Simultaneously Quantify Co-Loaded Dexamethasone and Melatonin PLGA Microspheres by HPLC-UV: Encapsulation Efficiency and In Vitro Release
Source: Pharmaceutics. 2022 Jan 26;14(2):288. doi: 10.3390/pharmaceutics14020288 (PMC8878730; doi:10.3390/pharmaceutics14020288)
Supplement: Supplementary file 1 [file pharmaceutics-14-00288-s001.zip › pharmaceutics-1418134-supplementary.pdf]

pharmaceutics

Supplementary Materials: Validation of a Rapid and Easy-to-Apply Method to Simultaneously Quantify Co-Loaded Dexamethasone and Melatonin PLGA Microspheres by HPLC-UV: Encapsulation Efficiency and In Vitro Release

Marco Brugnera, Marta Vicario-de-la-Torre, Vanessa Andrés-Guerrero, Irene Bravo-Osuna, Irene Teresa Molina-Martínez and Rocío Herrero-Vanrell

Table S1. Robustness results expressed in average recovery percentages of six WS-EE and WS-IVR regarding changes in different analysts performing the test. Level line referred to low (5 and 2.5 µg mL^{-1} respectively for procedures A and B), medium (20 and 7.5 µg mL^{-1} respectively for procedures A and B) and high (40 and 15 µg mL^{-1} respectively for procedures A and B) concentration levels.

		Procedure A						Procedure B					
		MEL			DX			MEL			DX		
	Level	Low	Medium	High	Low	Medium	High	Low	Medium	High	Low	Medium	High
Analyst 1	Average	98.76	100.41	100.58	101.45	97.75	98.62	102.08	101.94	101.59	101.64	100.95	100.48
	RSD %	1.16	0.75	0.35	1.01	0.48	0.36	0.72	0.45	0.98	1.07	1.51	0.86
Analyst 2	Average	100.08	101.31	100.54	102.14	98.87	98.58	99.14	100.36	98.97	97.85	101.67	100.75
	RSD %	0.93	0.73	0.16	0.89	0.43	0.16	0.76	0.22	0.42	1.36	1.05	0.60

RSD (%) MEL total procedure A	1.05			
RSD (%) DX total procedure A	1.76			
RSD (%) MEL total procedure B	1.42			
RSD (%) DX total procedure B	1.66			
Average tR (RSD %) MEL analyst 1	Procedure A	2.88 (0.21)	Procedure B	2.93 (0.24)
Average tR (RSD %) DX analyst 1		4.65 (0.14)		4.74 (0.17)
Average tR (RSD %) MEL analyst 2		2.88 (0.16)		2.94 (0.12)
Average tR (RSD %) DX analyst 2		4.66 (0.23)		4.73 (0.21)
Average pS (RSD %) MEL analyst 1		1.28 (1.11)		1.42 (0.67)
Average pS (RSD %) DX analyst 1		1.36 (1.26)		1.47 (1.25)
Average pS (RSD %) MEL analyst 2		1.30 (1.68)		1.40 (1.14)
Average pS (RSD %) DX analyst 2		1.36 (1.88)		1.45 (0.98)

Table S2. Robustness results expressed in average recovery percentages of six WS-EE and WS-IVR regarding changes in HPLC-UV equipment. Level line referred to low (5 and 2.5 µg mL-1 respectively for procedures A and B), medium (20 and 7.5 µg mL-1 respectively for procedures A and B) and high (40 and 15 µg mL-1 respectively for procedures A and B) concentration levels.

		Procedure A						Procedure B					
		MEL			DX			MEL			DX		
	Level	Low	Medium	High	Low	Medium	High	Low	Medium	High	Low	Medium	High
Equipment 1	Average	103.80	100.64	100.00	101.21	100.46	99.96	98.65	102.35	103.16	98.30	100.44	100.88
	RSD %	0.78	0.35	0.28	0.27	0.32	0.28	0.31	0.37	0.20	0.72	0.29	0.17
Equipment 2	Average	102.45	98.68	99.11	99.47	101.46	101.71	101.61	98.92	100.20	101.93	99.92	99.48
	RSD %	0.23	0.21	0.24	0.94	0.73	0.19	0.74	0.43	1.09	0.88	0.19	0.38

RSD (%) MEL total procedure A	1.86			
RSD (%) DX total procedure A	0.96			
RSD (%) MEL total procedure B	1.79			
RSD (%) DX total procedure B	1.24			
Average tR (RSD %) MEL equipment 1	Procedure A	2.87 (0.17)	Procedure B	2.96 (0.23)
Average tR (RSD %) DX equipment 1		4.66 (0.22)		4.77 (0.31)
Average tR (RSD %) MEL equipment 2		2.77 (0.13)		2.82 (0.22)
Average tR (RSD %) DX equipment 2		4.48 (0.21)		4.45 (0.17)
Average pS (RSD %) MEL equipment 1		1.25 (0.98)		1.44 (1.25)
Average pS (RSD %) DX equipment 1		1.31 (1.09)		1.41 (0.88)
Average pS (RSD %) MEL equipment 2		1.24 (1.78)		1.42 (1.66)
Average pS (RSD %) DX equipment 2		1.33 (1.18)		1.39 (0.74)
